# Controllable Layered Structures in Polyoxomolybdate-Surfactant Hybrid Crystals

**DOI:** 10.3390/ma3010158

**Published:** 2010-01-06

**Authors:** Takeru Ito, Toshihiro Yamase

**Affiliations:** 1 Chemical Resources Laboratory, Tokyo Institute of Technology, R1-21, 4259 Nagatsuta, Midori-ku, Yokohama 226–8503, Japan; 2 Department of Chemistry, School of Science, Tokai University, 1117 Kitakaname, Hiratsuka 259–1292, Japan; 3 (Inc.) MO Device, 2-14-10 Kanaiwa-higashi, Kanazawa 920–0335, Japan

**Keywords:** inorganic-organic hybrid crystal, polyoxometalate, surfactant

## Abstract

Inorganic-organic hybrid crystals containing α-octamolybdate (Mo_8_) or hexamolybdate (Mo_6_) were isolated by using hexadecyltrimethylammonium (C_16_) surfactant. The packing mode of the inorganic layers depended on a difference in the polyoxomolybdate molecular structure. The structure for both crystals consisted of alternate stacking of C_16_ organic bilayers and polyoxomolybdate inorganic layers with a periodicity of 24.4–24.6 Å. However, the C_16_-Mo_8_ crystals contained Mo_8_ monolayers, while the C_16_-Mo_6_ crystals contained Mo_6_ bilayers. These lattice structures for the polyoxometalate/organic hybrid will be designed by the molecular structures of polyoxometalate.

## 1. Introduction 

Crystalline layered materials have distinct anisotropy derived from two-dimensional strata of compounds, which often results in electronic conductivity, superconductivity, or intercalation [1,2,3]. The emergence of such properties is prompted by precise control of the layered structure such as the layer periodicity and/or component arrangement. Inorganic-organic hybrids [4] are more structurally controllable than purely inorganic compounds owing to organic components, and have potential for the construction of functionalized crystalline layered materials. Conductive hybrid crystals composed of organic molecules and inorganic anions have been reported [5,6]. 

Surfactant molecules are an effective organic component as a structure-directing reagent for lamellar structures [7,8]. The layer distance can be controlled by changing the length of long alkyl chains. Polyoxometalate anions with various physicochemical properties are promising candidates for an inorganic component [9,10,11], and can be selected to design the composition, functions, and even structures of hybrid layered crystals. Several hybrid materials [12,13,14,15,16,17] and hybrid layered crystals [18,19,20,21,22,23] containing polyoxometalates and surfactants have been prepared to date. 

Here, we report the controllable synthesis of polyoxomolybdate hybrid layered crystals containing hexadecyltrimethylammonium (C_16_). Two types of crystals, [(C_16_H_33_)N(CH_3_)_3_]_4_[α-Mo_8_O_26_] (**1**) and [(C_16_H_33_)N(CH_3_)_3_]_2_[Mo_6_O_19_] (**2**), had different crystal packings, which will be induced by the molecular structures of polyoxomolybdate. 

## 2. Results and Discussion 

The syntheses of **1** and **2** are based on the procedure for the preparation of tetrabutylammonium hexamolybdate [24]. However, a pale yellow precipitate obtained after adding C_16_Br to Na_2_MoO_4_ solution (see Experimental) is a mixture of Mo_6_O_19_^2-^ (Mo_6_) and α-Mo_8_O_26_^4-^ (Mo_8_) anions indicated by IR spectra (not shown). The pale yellow color of the precipitate also suggests the presence of Mo_6_ (yellow) and Mo_8_ (colorless). The recrystallization of this mixture from hot acetonitrile gives pure crystals of **1**, which is less soluble in acetonitrile than **2**. The remaining pale yellow supernatant contains the Mo_6_ anion, from which pure crystals of **2** can be obtained by evaporating or cooling. 

**Figure 1:**
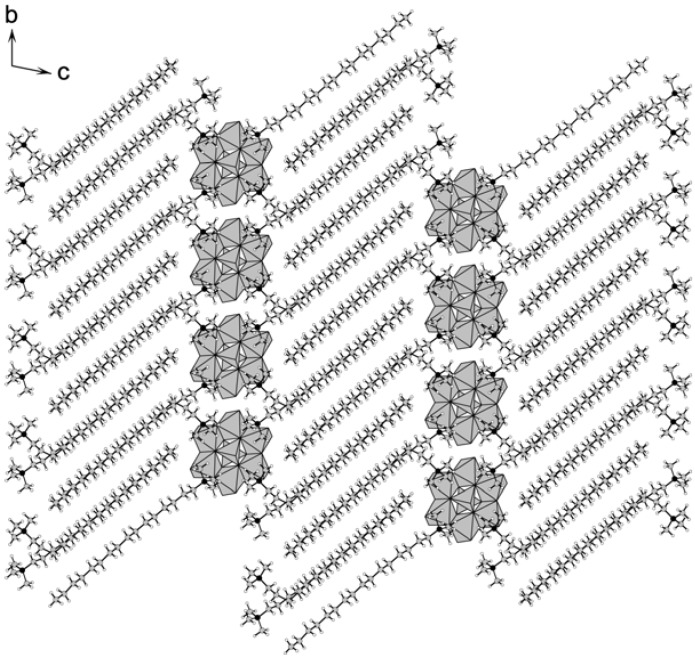
Crystal packing of **1** (C: grey, N: black, H: white; Mo_8_ anions in grey polyhedra).

The crystal packing of **1** consists of alternating inorganic monolayers of α-type Mo_8_ and organic bilayers of C_16_ cations (Figure 1). This manner of packing is the same as those of other polyoxometalate-surfactant hybrid crystals reported to date [18,19,20,21,22,23]. The periodicity between the inorganic and organic layers is 24.4 Å. The hexadecyl chains of C_16_ interdigitate in the C_16_ bilayers, and the hydrophilic heads of C_16_ insert into the Mo_8_ monolayers with a depth of 3.04 Å, which is similar to other polyoxometalate hybrid crystals containing surfactants with single alkyl chain [18,21,22,23]. 

**Figure 2:**
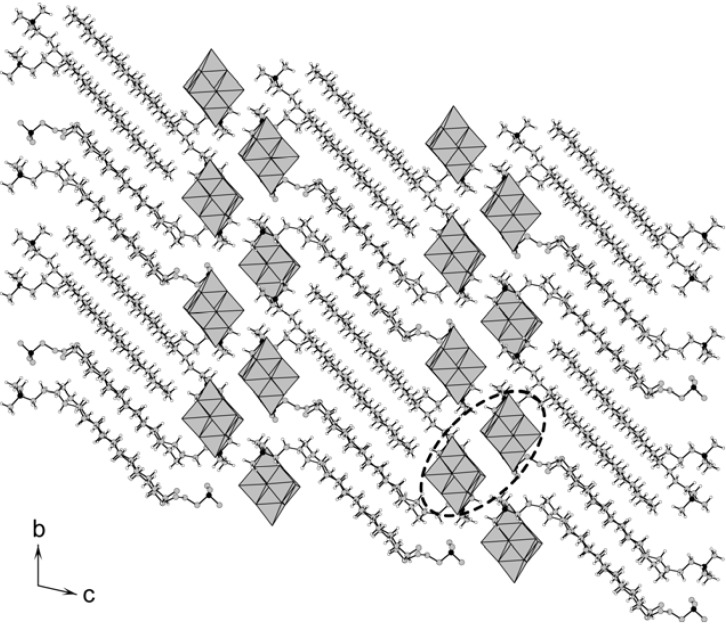
Crystal packing of **2** (C: grey, N: black, H: white; Mo_6_ anions in grey polyhedra). Disordered atoms were omitted for clarity. The broken line indicates a “dimer-like” structure of the Mo_6_ anions (see text).

The lattice structure of **2** also consists of alternating inorganic layers and organic interdigitated bilayers of C_16_ with a periodicity of 24.6 Å, similar to that of **1**. However, the inorganic layer of Mo_6_ is a bilayer, quite different from **1** and other polyoxometalate-surfactant crystals [18,19,20,21,22,23]. The hydrophilic heads of C_16_ completely insert into the Mo_6_ bilayers. The different packings of polyoxomolybdate for **1** and **2** will be induced by the difference in the molecular structures of Mo_8_ and Mo_6_. The distance between the nearest Mo_6_ anions is 2.28 Å, and the two adjacent Mo_6_ anions form a “dimer-like” structure (indicated by the broken line in Figure 2). The Mo_6_ “dimers” arrange two-dimensionally parallel to the *ab* plane, considered to result in the formation of the Mo_6_ bilayer. **2** is the first example which contains polyoxometalate bilayers in the polyoxometalate-surfactant hybrid crystal. Changing the molecular structure of polyoxometalate as well as surfactant can control the layered structure of the hybrid crystals. 

Both **1** and **2** have C-H···O hydrogen bonds [25,26,27,28,29,30] at the interface between the polyoxomolybdate and C_16_ layers. The C···O distances of the hydrogen bonds are mainly 3.3–3.6 Å (mean value: 3.52 Å) for **1** and 3.3–4.0 Å (mean value: 3.54 Å) for **2**, respectively. Most hydrogen bonds are formed between oxygens of polyoxomolybdate and the hydrophilic head of C_16_ (*i.e.,* methyl or methylene groups connected to nitrogen). The hydrogen bonds as well as electrostatic interaction between polyoxomolybdate and C_16_ layers are considered to stabilize the layered structures of **1** and **2**. 

**Table 1:** Crystallographic data for **1** and **2**.

	1	2
**Chemical formula**	C_76_H_168_N_4_Mo_8_O_26_	C_38_H_84_N_2_Mo_6_O_19_
**Formula weight**	2321.66	1448.71
**Crystal system**	triclinic	triclinic
**Space group**	*P*$\bar{\text{1}}$ (No.2)	*P*$\bar{\text{1}}$ (No.2)
***a* (Å)**	9.958(8)	9.911(8)
***b* (Å)**	11.149(3)	22.34(3)
***c* (Å)**	24.95(2)	25.58(3)
***α* (°)**	98.06(4)	102.78(4)
***β* (°)**	94.828(7)	99.12(3)
***γ* (°)**	115.66(4)	91.19(4)
***V* (Å^3^)**	2439(3)	5444(10)
***Z***	1	4
***ρ*_calcd_ (g cm^-3^)**	1.580	1.768
***T* (K)**	173	173
***μ*(Mo Kα) (mm^-1^)**	1.062	1.407
**No. of reflections measured**	21313	48956
**No. of independent reflections**	10383	22835
**No. of parameters**	515	594
***R*_1_ (*I* > 2*σ*(*I*))**	0.0513	0.0642
***wR*_2_ (all data)**	0.0921	0.1748

## 3. Experimental 

### 3.1. Syntheses

Compounds **1** and **2** were synthesized by a modified literature procedure [24]. To 10 mL of aqueous solution of Na_2_MoO_4_•2H_2_O (2.5 g, 10.3 mmol) was added 7 M HCl (2.9 mL, 20.9 mmol) with vigorous stirring. After 1 min, a water/ethanol (15 mL, 2:1 (v/v)) solution of C_16_Br (1.37 g, 3.8 mmol) was added to form a pale yellow precipitate. This suspension was heated at 60–80 ºC for 90 min with stirring, then filtered and dried with suction. Recrystallization of the crude product from hot acetonitrile gave colorless plates of **1**, and the remaining pale yellow supernatant was air-dried to obtain yellow plates of **2**. Data for **1**: Anal. Calcd. for C_76_H_168_N_4_Mo_8_O_26_: C, 39.3; H, 7.3; N, 2.4%. Found: C, 39.4; H, 6.9 N, 2.5%. IR (KBr disk): 952 (m), 917 (s), 859 (m), 806 (s), 720 (w), 668 (m), 554 (w) cm^-1^. Data for **2**: Anal. Calcd. for C_38_H_84_N_2_Mo_6_O_19_: C, 31.5; H, 5.8; N, 1.9%. Found: C, 31.5; H, 5.7 N, 2.0%. IR (KBr disk): 964 (s), 799 (s) cm^-1^. 

### 3.2. Crystallography

All measurements were made on a Rigaku RAXIS RAPID imaging plate diffractometer with graphite monochromated Mo-Kα radiation (*λ* = 0.71075 Å). Numerical absorption correction was performed for **1**, and empirical absorption correction was performed for **2**. The both structures were solved by direct methods (SHELXS-97) and refined (SHELXL-97) with *SHELX-97* [31] and *CrystalStructure* [32] software packages. 

In the refinement procedure for **1**, all non-hydrogen atoms were refined anisotropically, and the hydrogen atoms on C atoms were located in calculated positions. For **2,** Mo atoms were refined anisotropically, while other non-hydrogen atoms were refined isotropically utilizing suitable restraints of the N-C and C-C distances. Some C atoms were disordered. The hydrogen atoms on C atoms were located in calculated positions, while several hydrogen atoms relevant to the disordered C atoms were not included in the refinement. 

## 4. Conclusions 

We have synthesized two polyoxometalate hybrid crystals of [(C_16_H_33_)N(CH_3_)_3_]_4_[α-Mo_8_O_26_] (**1**) and [(C_16_H_33_)N(CH_3_)_3_]_2_[Mo_6_O_19_] (**2**) by using one kind of surfactant. The layered structures are formed by the alternate stacking of polyoxomolybdate inorganic layers and C_16_ organic bilayers. The packing manner of Mo_8_ in **1** and Mo_6_ in **2** is different, which reveals that the lattice structure can be designed in the polyoxometalate/surfactant hybrids by the molecular structure of polyoxometalate.

## Supplementary Materials

Supplementary File 1

Supplementary File 2
